# *C9ORF72* Repeat Expansion Affects the Proteome of Primary Skin Fibroblasts in ALS

**DOI:** 10.3390/ijms221910385

**Published:** 2021-09-27

**Authors:** Marta Lualdi, Adeena Shafique, Edoardo Pedrini, Luisa Pieroni, Viviana Greco, Massimo Castagnola, Giorgia Cucina, Lucia Corrado, Alice Di Pierro, Fabiola De Marchi, Lara Camillo, Claudia Colombrita, Marianna D’Anca, Tiziana Alberio, Sandra D’Alfonso, Mauro Fasano

**Affiliations:** 1 Biochemistry and Functional Proteomics Laboratory, Department of Science and High Technology, Center of Bioinformatics and Center of Neuroscience, University of Insubria, I-21052 Busto Arsizio, Italy; ashafique@uninsubria.it (A.S.); edoardo.pedrini@uninsubria.it (E.P.); gcucina@studenti.uninsubria.it (G.C.); tiziana.alberio@uninsubria.it (T.A.); mauro.fasano@uninsubria.it (M.F.); 2 Proteomics and Metabolomic Laboratory, Experimental Neuroscience Department, S. Lucia Foundation IRCCS, I-00168 Rome, Italy; l.pieroni@hsantalucia.it (L.P.); maxcastagnola@outlook.it (M.C.); 3 Department of Basic Biotechnological Sciences, Intensivological and Perioperative Clinics, Università Cattolica del Sacro Cuore, I-00168 Rome, Italy; viviana.greco@unicatt.it; 4 Molecular and Genomic Diagnostics Unit, Fondazione Policlinico Universitario “A. Gemelli” IRCCS, I-00168 Rome, Italy; 5 Department of Health Sciences, University of Eastern Piedmont “A. Avogadro”, I-28100 Novara, Italy; lucia.corrado@med.uniupo.it (L.C.); alice.dipierro@uniupo.it (A.D.P.); lara.camillo@uniupo.it (L.C.); sandra.dalfonso@med.uniupo.it (S.D.); 6 Department of Translational Medicine, University of Eastern Piedmont “A. Avogadro”, I-28100 Novara, Italy; fabiola.demarchi@uniupo.it; 7 Department of Neurology and ALS Centre, “Maggiore della Carità” Hospital, I-28100 Novara, Italy; 8 Department of Neurology-Stroke Unit and Laboratory of Neuroscience, Istituto Auxologico Italiano IRCCS, I-20149 Milan, Italy; claudia.colombrita@unimi.it; 9 Neurodegenerative Disease Unit, Fondazione Ca’ Granda IRCCS, Policlinico Hospital, I-20122 Milan, Italy; marianna.danca@policlinico.mi.it

**Keywords:** *C9ORF72*, amyotrophic lateral sclerosis, proteomics, skin fibroblasts, PPI network, functional enrichment analysis

## Abstract

Amyotrophic lateral sclerosis (ALS) is a neurodegenerative disease characterized by progressive degeneration of the corticospinal motor neurons, which ultimately leads to death. The repeat expansion in chromosome 9 open reading frame 72 (*C9ORF72*) represents the most common genetic cause of ALS and it is also involved in the pathogenesis of other neurodegenerative disorders. To offer insights into *C9ORF72*-mediated pathogenesis, we quantitatively analyzed the proteome of patient-derived primary skin fibroblasts from ALS patients carrying the *C9ORF72* mutation compared with ALS patients who tested negative for it. Differentially expressed proteins were identified, used to generate a protein-protein interaction network and subjected to a functional enrichment analysis to unveil altered molecular pathways. ALS patients were also compared with patients affected by frontotemporal dementia carrying the *C9ORF72* repeat expansion. As a result, we demonstrated that the molecular pathways mainly altered in fibroblasts (e.g., protein homeostasis) mirror the alterations observed in *C9ORF72*-mutated neurons. Moreover, we highlighted novel molecular pathways (nuclear and mitochondrial transports, vesicle trafficking, mitochondrial bioenergetics, glucose metabolism, ER-phagosome crosstalk and Slit/Robo signaling pathway) which might be further investigated as *C9ORF72*-specific pathogenetic mechanisms. Data are available via ProteomeXchange with the identifier PXD023866.

## 1. Introduction

The GGGGCC hexanucleotide repeat expansion of chromosome 9 open reading frame 72 (*C9ORF72*) gene is the most common genetic mutation in amyotrophic lateral sclerosis (ALS), where it accounts for approximately 40% and 6% of the familial and sporadic cases, respectively [1]. ALS is a neurodegenerative disorder characterized by progressive degeneration of the corticospinal motor neurons leading to paralysis and ultimately death from respiratory failure [2]. Most patients die within 3–10 years after disease onset and available treatments are only palliative, thus there is an urgent need to investigate the molecular processes underlying early pathogenesis by using omics approaches; this will suggest novel biomarkers and therapeutic interventions.

The GGGGCC hexanucleotide repeat is located between two five-prime non-coding exons of the *C9ORF72* gene resulting in 5′-UTR of the transcript [3]. Affected individuals usually carry one normal and one expanded allele. The number of repeats in the general population ranges between 2 and 23, whereas pathological condition results with a minimum of 30 repeats and the number can reach thousands [4]. As in many other non-coding repeat expansion diseases, the main underlying pathogenetic mechanism involves an RNA gain-of-function: the aberrant transcripts produced by the expanded allele are retained in the nucleus and accumulate in RNA foci. The accumulation of expanded transcripts as nuclear RNA foci has been demonstrated in the frontal cortex and spinal cord of patients carrying the *C9ORF72* repeat expansion [3]. RNA foci can sequester and/or alter the function of several RNA-binding proteins, which results in changes in expression and RNA processing of other genes [5]. In addition to this, two other pathogenetic mechanisms have been proposed: (i) toxicity due to the aggregation of dipeptide repeats produced by non-ATG-mediated RNA translation; and (ii) loss-of-function of the *C9ORF72* protein by means of haploinsufficiency [6]. In this context, some recent studies have shed light on the function of *C9ORF72* protein, which acts as a guanine nucleotide exchange factor (GEF) for both Rab- and Rho-GTPases, thus suggesting a function in endocytosis, cytoskeleton modulation and autophagy [7,8,9]. *C9ORF72* also regulates actin dynamics [10] and stress granules formation [11]. Several metabolic, proteostasis and neuronal processes are known to be impaired in *C9ORF72*-mediated pathogenesis, including lysosomal function, ubiquitin-proteasome system (UPS), unfolded protein response (UPR), axonal transport and immune function [12]. In other words, both loss- and gain-of-function mechanisms are implicated in *C9ORF72*-mediated pathogenesis. The gain-of-function ones support the formation of toxic aggregates (both RNA and proteins), whereas the loss-of-function mechanisms mainly impair vesicle trafficking and autophagy.

Disease-causing expansions in *C9ORF72* were first reported in 2011 in ALS patients and were then found in other neurodegenerative disorders. Indeed, the *C9ORF72* repeat expansion represents the most frequent genetic mutation also present in patients affected by frontotemporal dementia (FTD), which can contribute to explaining the partial clinical overlap between ALS and FTD [3,13,14]. Indeed, although the main clinical manifestation of FTD is dementia due to the degeneration of frontal and temporal lobes [15], in some FTD patients, motor impairment is also prominent [16]. On the other hand, ALS patients can also develop cognitive impairments that are characteristic of FTD [17]. Moreover, a possible association with Parkinson’s disease (PD), atypical parkinsonian syndromes, Alzheimer’s disease (AD) and Huntington’s disease (HD) has recently been reported, where the *C9ORF72* repeat expansion has been proposed as either a causative or susceptibility factor [18,19]. In this frame, elucidating the molecular mechanisms hampered by the presence of the *C9ORF72* repeat expansion will be crucial for the identification of candidate targets for therapeutic interventions and/or diagnosis and prognosis in several neurodegenerative disorders.

Herein, the results of an in-depth mass-spectrometry-based proteomics analysis of primary skin fibroblasts in ALS patients are reported. The study was designed to compare ALS patients carrying the *C9ORF72* repeat expansion with ALS patients who tested negative for the expansion, with the aim to highlight a proteomics signature associated with the presence of this mutation and possibly unveil *C9ORF72*-related pathogenetic mechanisms in ALS. Combining proteomics and systems biology analyses led us to identify a list of differentially expressed proteins and generate protein-protein interaction (PPI) networks to highlight molecular pathways that were significantly altered by comparing the two groups of ALS patients. Eventually, ALS patients were compared with a small cohort of *C9ORF72*-mutated FTD patients.

## 2. Results

### 2.1. Experimental Design: Exploring the Impact of C9ORF72 Repeat Expansion on the Proteome of Primary Skin Fibroblasts of ALS Patients

Primary skin fibroblast cell lines were obtained from ALS patients carrying the *C9ORF72* repeat expansion (ALS_C9; *n* = 8) and ALS patients who tested negative for the *C9ORF72* repeat expansion (ALS_N; *n* = 8). Among ALS_N, six patients also tested negative for the remaining three main ALS genes (*SOD1*, *TARDBP* and *FUS*) while two patients carried a mutation in *SOD1*.

**Figure 1:**
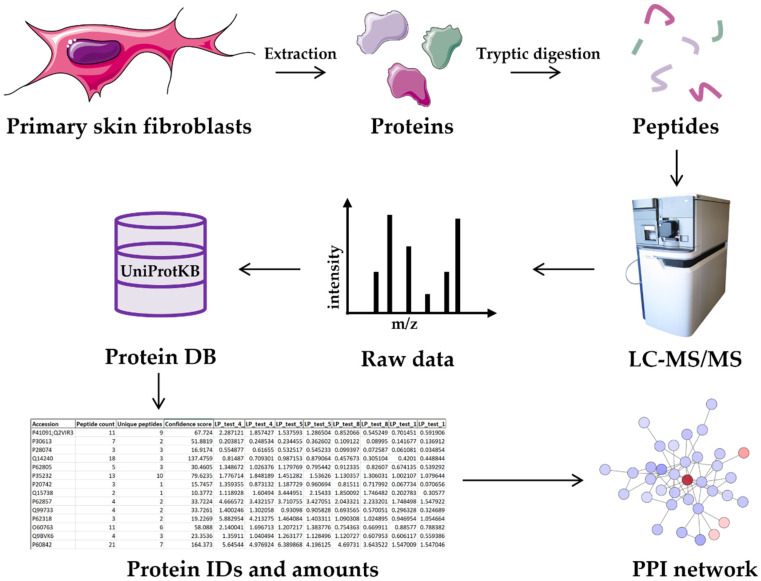
Experimental workflow. Proteins extracted from primary skin fibroblasts of all patients were digested with trypsin and the peptides were analyzed by LC-MS/MS. The resulting raw data were used to query the UniProt database and proteins were identified. The list of identified and quantified proteins was used for subsequent analyses, including differential expression analysis, PPI networks generation and functional enrichment analysis (cell and proteins images from: https://bioicons.com/ (accessed on 9 July 2021)).

The proteome of fibroblasts was quantitatively analyzed by LC-MS/MS. A total of 1198 quantifiable proteins were identified with no missing values (Supplementary Table S1). The raw quantification of the proteins was normalized by quantile before proceeding with the identification of the targets of interest (Figure 1).

**Figure 2:**
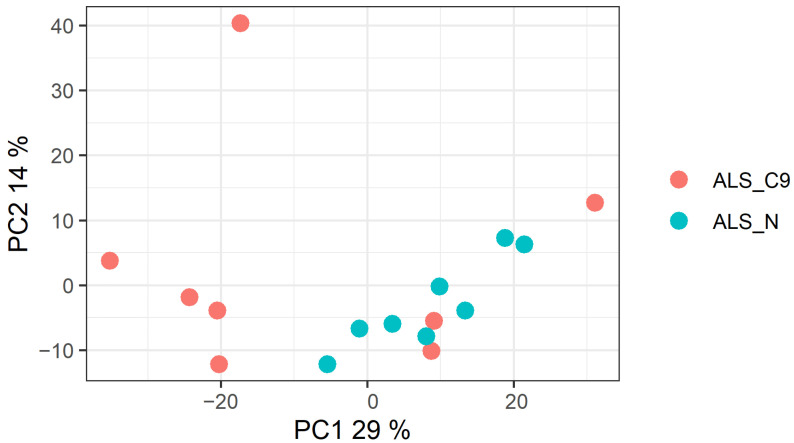
PCA comparing ALS_C9 and ALS_N patients’ proteome. The PCA analysis was performed considering all quantified proteins in fibroblasts from patients included in the two groups (*n* = 16). Each dot represents a patient. Pink: ALS_C9. Light blue: ALS_N.

At first, we decided to explore the dataset in order to identify the main source of variation. We therefore performed a principal component analysis (PCA) over the normalized dataset and, interestingly, we identified that PC1 was mainly separating the samples by condition (Figure 2). Three ALS_C9 samples were segregating with the main bulk of ALS_N samples; therefore, we decided to check the disease history and the clinical anamnesis of these three ALS_C9 patients, looking for the possible presence of some exclusion criteria. We noticed that one of them was affected by dementia, while a family history of FTD was reported for the other two. We concluded that the evidence provided by the PCA and the clinical phenotype was enough to label the three samples as outliers; therefore, we decided to exclude these three patients from the ALS_C9 group, thus keeping five ALS_C9 patients for further analyses.

### 2.2. Differential Expression Analysis

**Figure 3:**
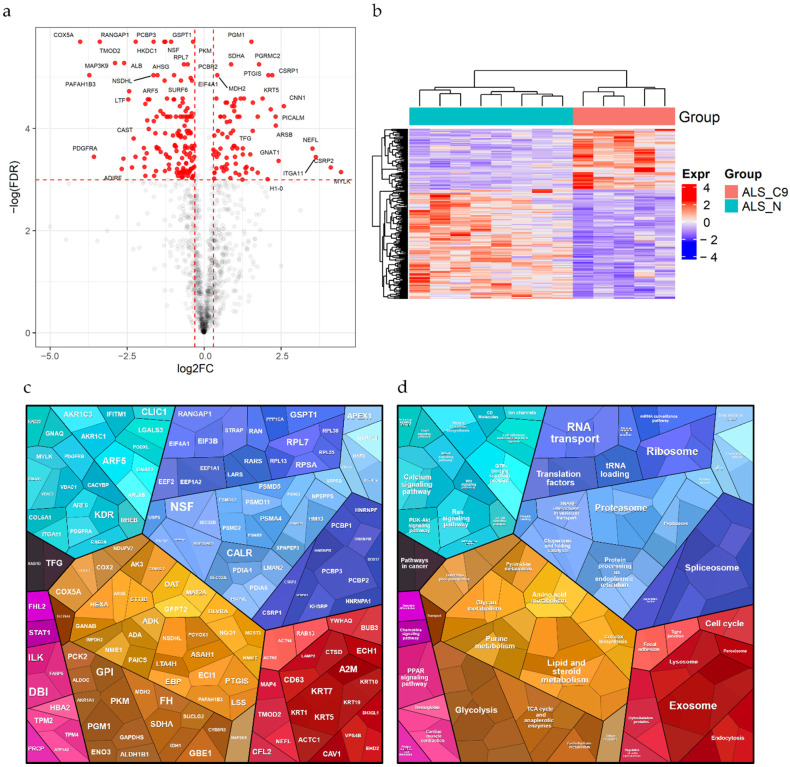
Differential expression analysis (ALS_C9 vs. ALS_N). (**a**) Volcano plot showing all quantified proteins distributed by FC and FDR in log scale (ALS_C9 over ALS_N). Red dashed lines represent thresholds set for significance. Red dots represent differentially expressed proteins (*n* = 216). (**b**) Heatmap demonstrating the separation of all patients into two clusters (corresponding to the ALS_C9 and the ALS_N groups) based on the expression levels of the 216 significant proteins (rows). (**c**,**d**) Voronoi plots showing the differentially expressed proteins grouped and colored by their function. Areas are proportional to the *p* values. Turquoise: environmental information processing (e.g., calcium signaling pathways, Ras signaling pathway, GTP-binding signaling proteins). Blue: genetic information processing (e.g., RNA transport, ribosome, proteasome, spliceosome). Red: cellular processes (e.g., exosome, cell cycle, endocytosis). Yellow: metabolism (e.g., glycolysis, lipid and steroid metabolism, TCA cycle and anaplerotic enzymes). Pink: signaling (e.g., PPAR signaling pathway). Grey: human diseases (pathways in cancer).

A differential expression analysis of all proteins quantified by LC-MS/MS (FC = ALS_C9 over ALS_N) was then performed to determine the effect of *C9ORF72* repeat expansion on the proteome of skin fibroblasts of ALS patients. Thresholds for significance have been defined as absolute log_2_(FC) > 0.3 and adjusted *p* value < 0.05 (Figure 3a). Proteins with significantly altered levels between the two groups were 216 (Supplementary Table S2), of which 77 proteins were upregulated and 139 proteins were downregulated in ALS_C9 patients compared with ALS_N. To verify the goodness of the arbitrary thresholds imposed for filtering the significant proteins, we checked the expression levels of this panel of significant proteins across all samples. As expected, all patients were correctly grouped into two separate clusters (Figure 3b). Differentially expressed proteins were also annotated based on the cellular process of their components and rendered as a Voronoi plot (Figure 3c,d). The results showed that the significant proteins were involved in different cellular processes, such as RNA processing, protein homeostasis and several signaling pathways.

### 2.3. Functional Enrichment Analyses

**Figure 4:**
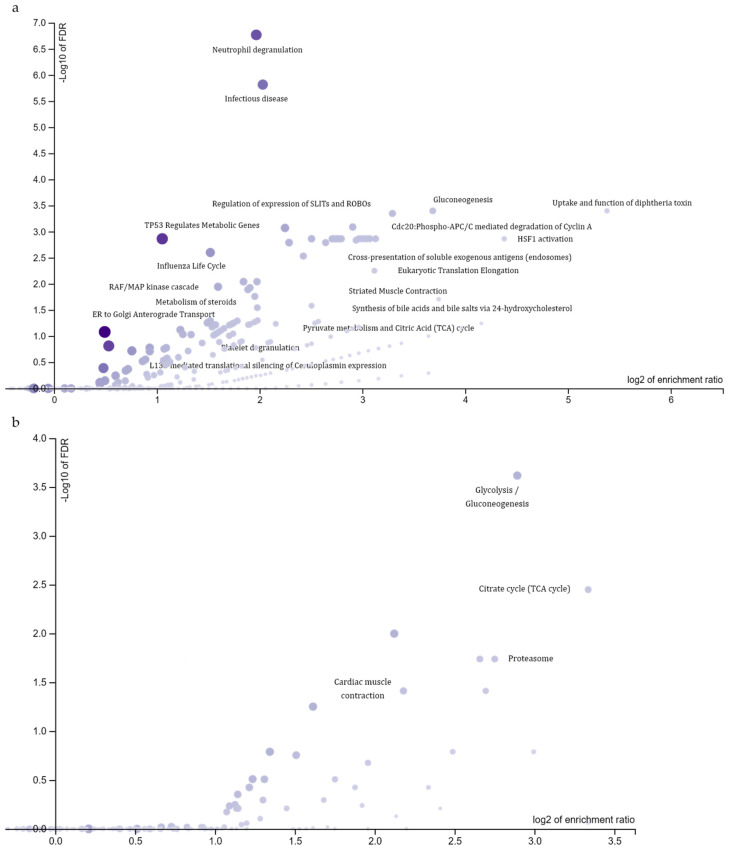
Functional enrichment analysis of differentially expressed proteins. Volcano plots displaying the most significantly enriched terms distributed by enrichment ratio and FDR in log scale. (**a**) Reactome database, nineteen terms labelled. (**b**) KEGG database, four terms labelled.

The protein annotation described above was not yet contextualized with the global proteome. Indeed, it did not represent a functional enrichment analysis; rather it only assigned a functional class to all differentially expressed proteins. Thus, the protein list of differentially abundant proteins (*n* = 216) was subjected to an over-representation analysis (ORA) to highlight the molecular pathways specifically enriched by the presence of the *C9ORF72* repeat expansion in fibroblasts of ALS patients (Figure 4).

By using Reactome as pathway database, the functional enrichment analysis highlighted 141 enriched pathways (Supplementary Table S3), then grouped in nineteen terms after the application of “affinity propagation” as redundancy reduction method (Figure 4a). The most significantly enriched terms were: (i) neutrophil degranulation (R-HSA-6798695; FDR = 1.7 × 10^−7^), which included proteasome 26S subunits, glycolytic enzymes, proteins involved in actin filament dynamics, translation elongation factors, members of the HSP90 family, late endosomal/lysosomal adaptors and lysosome-associated proteins; (ii) infectious disease (R-HSA-5663205; FDR = 1.5 × 10^−6^), which included proteasome 26S subunits, members of the HSP90 family, translation elongation factors, ribosomal proteins, RAN and RAN-GTPase-activating proteins; (iii) gluconeogenesis (R-HSA-70263; FDR = 4.0 × 10^−4^), which included several glycolytic enzymes and members of the mitochondrial solute carrier family. Similarly, by using the KEGG database, a total number of eight enriched pathways have been identified (Supplementary Table S4), reduced to four terms by applying the “affinity propagation” method for redundancy reduction (Figure 4b). The most significant in terms of FDR were: (i) glycolysis/gluconeogenesis (hsa00010; FDR = 2.4 × 10^−4^), including several metabolic enzymes; (ii) citrate cycle (TCA cycle) (hsa00020; FDR = 3.6 × 10^−3^); (iii) proteasome (hsa03050; FDR = 1.8 × 10^−2^), including mainly proteasome 26S subunits.

### 2.4. PPI Network Generation and Analysis

**Figure 5:**
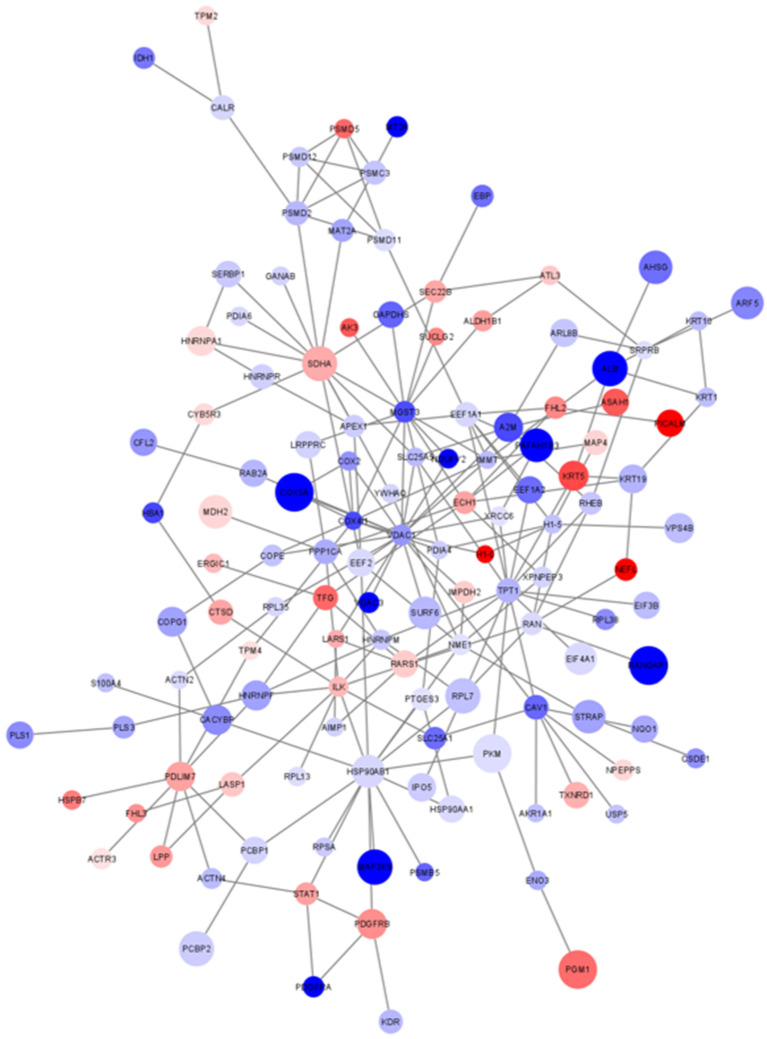
Main component of the PPI network of differentially expressed proteins. Each node represents one protein (*n* = 129). Node color is associated with the FC. Blue: proteins downregulated in ALS_C9 vs. ALS_N; red: proteins upregulated in ALS_C9 vs. ALS_N. Node size is proportional to the −log_10_(adjusted *p* value): the larger the size, the lower the protein-associated *p* value.

In order to better highlight connections among proteins and to perform a further functional enrichment analysis, a PPI network was then generated using the list of significantly altered proteins. The whole list of differentially expressed proteins (*n* = 216) generated a network including a main component of connected nodes (*n* = 129, *p* value < 1 × 10^−4^; Supplementary Table S5; Figure 5) and some unconnected nodes (*n* = 87), which were excluded from subsequent analysis.

An ORA was then performed using the list of proteins included in the main component as input list and Reactome as pathway database. The analysis highlighted 161 enriched pathways (Supplementary Table S6). After redundancy reduction, the most significant enriched terms were: (i) infectious disease (R-HSA-5663205; FDR = 7.6 × 10^−8^), which included translation elongation factors, members of the HSP90 family, proteasome 26S subunits, ribosomal proteins, RAN and RAN-GTPase-activating proteins, the solute carrier family 25 member 5 (SLC25A5) and the vacuolar protein sorting 4 homolog B (VPS4B); (ii) regulation of ornithine decarboxylase (ODC) (R-HSA-350562; FDR = 4.1 × 10^−4^), which included proteasome 26S subunits; (iii) translation (R-HSA-72766; FDR = 4.1 × 10^−4^), including translation initiation and elongation factors, ribosomal proteins, some aminoacyl-tRNA synthetases and SRP receptor subunits.

**Figure 6:**
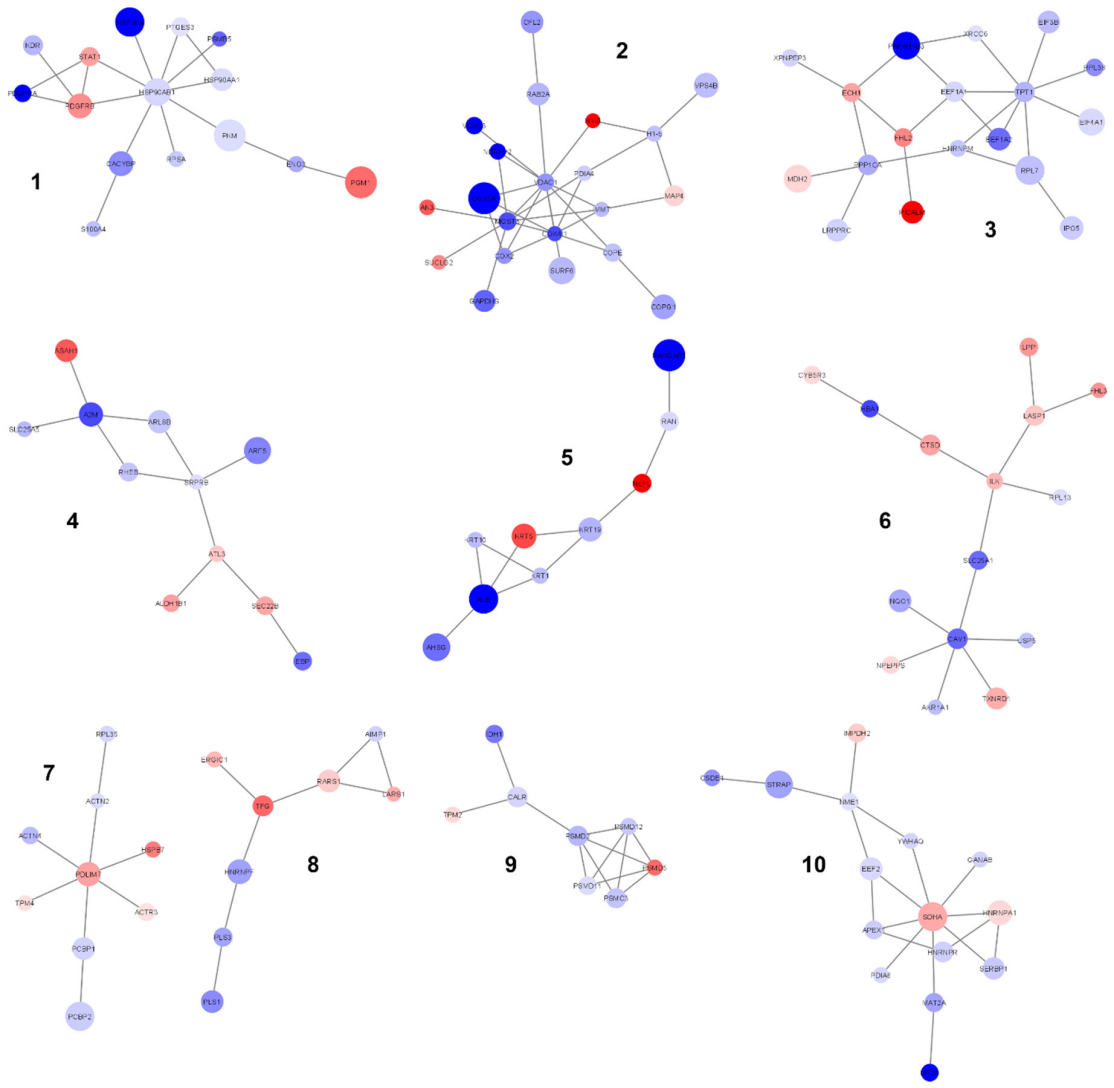
Sub-clusters obtained by the segmentation of the main component. Ten sub-clusters are shown. Node color is associated with the FC. Blue: proteins downregulated in ALS_C9 vs. ALS_N; red: proteins upregulated in ALS_C9 vs. ALS_N. Node size is proportional to the statistical significance.

The main component was then segmented into sub-clusters by the GLay community-finding algorithm to find out highly interconnected proteins and possibly unveil some pathways that were otherwise hidden in the main component. Ten sub-clusters were generated by the segmentation of the main network (Supplementary Table S7; Figure 6).

Each sub-cluster was further analyzed by ORA and results are listed in Supplementary Table S8. Significantly enriched functional terms were highlighted in seven out of ten sub-networks; some of them had already emerged from the analysis of the main component while some others were newly identified. As examples, sub-cluster #1 was enriched in proteins belonging to the heat shock protein family and proteins involved in signal transduction (e.g., STAT1), sub-cluster #2 unveiled TCA cycle, respiratory electron transport and ATP synthesis (COX2, COX5A, VDAC1), sub-cluster #3 further highlighted translation (ribosomal proteins RPL7 and RPL38; initiation and elongation factors EIF3B, EIF4A1, EEF1A1, EEF1A2), sub-cluster #8 highlighted tRNA aminoacylation (Leucyl- and Arginyl-tRNA synthetases LARS and RARS) and sub-cluster #9 unveiled the ER-phagosome pathway. No significant functional terms emerged in sub-clusters #4, #7 and #10.

### 2.5. Comparison between ALS and FTD as Both Are C9ORF72-Associated Diseases

**Figure 7:**
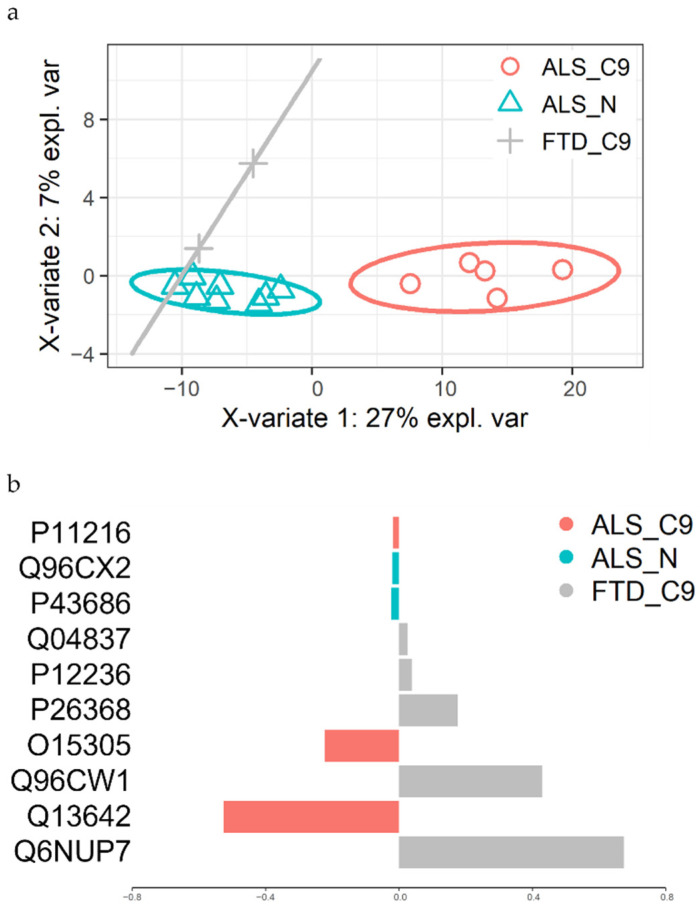
sPLS-DA analysis. Supervised multivariate analysis was performed considering all quantified proteins in fibroblasts from patients included in the three groups. (**a**) Scatter plot displaying the distribution of all patients. Pink: ALS_C9; *n* = 5. Light blue: ALS_N; *n* = 8. Grey: FTD_C9; *n* = 2. (**b**) Proteins mainly contributing to the separation along the PC2, thus discriminating FTD from all ALS patients.

The *C9ORF72* repeat expansion is also the main causative genetic alteration in FTD. Since we had the opportunity to collect and analyze skin fibroblast samples also from two *C9ORF72*-mutated FTD patients (FTD_C9), we decided to perform a preliminary assessment to compare the proteome among the three groups (ALS_C9, ALS_N and FTD_C9). To this end, a supervised multivariate analysis was performed in order to verify whether the composition of the proteome of fibroblast cells was able to discriminate between the three groups of patients. Specifically, a sparse partial least squares discriminant analysis (sPLS-DA) was applied, including all proteins quantified in all samples. As shown in Figure 7, the three groups were actually separated: ALS_C9 separated by ALS_N through the PC1 and the two FTD patients separated by all ALS through the PC2. Interestingly, by looking into the features whose contribution was high in the second component, we found two mitochondrial proteins (Q04837: single-stranded DNA-binding protein; P12236: ADP/ATP translocase 3) and one protein involved in vesicle dynamics (Q96CW1: AP-2 complex subunit mu).

## 3. Discussion

In this study, we showed the results of a proteomics analysis performed on primary skin fibroblast cell lines from ALS patients with the aim of unveiling pathogenetic mechanisms related to the *C9ORF72* repeat expansion. This genetic variant is the main causal factor of familial forms of ALS and other neurodegenerative diseases; nevertheless it is currently unclear which pathogenetic mechanisms are implied, which molecular pathways are mainly altered and how the disease course is affected by the mutation, eventually leading to a broad spectrum of clinical phenotypes. Here, by using a proteomics approach coupled to a network-based systems biology analysis, we shed some light on the molecular mechanisms underlying *C9ORF72*-mediated pathogenesis.

Investigating molecular mechanisms in neurodegenerative diseases is always complicated by the experimental unavailability of the actual diseased cell type, i.e., patients’ neurons. Thus, one of the trickiest aspects of neurodegeneration research is to identify and use a proper patient-derived cell model for experimental purposes, which mirrors neuronal cell alterations. As the experimental model in our study, we used primary skin fibroblast cell lines cultured from patients’ skin biopsies. This model presents multiple advantages: (i) these cells are easily available, robust and serve as an accessible source of patient-derived proliferating cells; (ii) they can be sub-cultured, maintained for longer durations and used to generate pluripotent stem cells; and (iii) they are effective for studying disease pathogenesis even though they do not represent the actual cells in which the pathogenetic process occurs. The use of this cellular model has been extensively reported in neurodegeneration literature in order to unveil molecular mechanisms and/or candidate biomarkers [20,21,22,23,24,25,26,27]. However, a thorough characterization of ALS fibroblast cells as model in the context of the *C9ORF72* repeat expansion is currently lacking in literature.

The study design included two groups of patients sharing the disease phenotype though bearing a different genetic background (ALS_C9 and ALS_N). In the context of this comparison, the total proteome of fibroblasts from ALS patients carrying the *C9ORF72* repeat expansion was analyzed against that of ALS patients who tested negative for the *C9ORF72* repeat expansion, including patients also negative for the remaining three main ALS genes (*SOD1*, *TARDBP* and *FUS*) and patients carrying a mutation in *SOD1*. Differentially expressed proteins emerging from this setup are central to highlight which molecular mechanisms are specifically affected by the presence of the *C9ORF72* repeat expansion in ALS patients. The functional enrichment analysis performed on the list of differentially expressed proteins unveiled glucose metabolism and protein homeostasis (translation, folding and degradation) as main altered pathways. This is consistent with the fact that the *C9ORF72* repeat expansion has a strong impact on general proteostasis due to the accumulation of aberrant transcripts and proteins [28,29]. Moreover, the enrichment of the “protein folding” pathway in ALS patients carrying the *C9ORF72* repeat expansion in comparison to ALS patients negative for the *C9ORF72* repeat expansion also emerged from a recent and extensive RNA sequencing study performed on frontal cortex tissue [30]. Notably, we noticed that several proteins involved in the translation process, especially ribosomal proteins, were strongly downregulated in ALS_C9 if compared with ALS_N. This evidence suggests a general inhibition/blockade of protein synthesis in ALS_C9, which could represent a counteractive response to the accumulation of toxic protein aggregates. Collectively, the results of our differential expression and functional enrichment analyses clearly support the use of skin fibroblasts as a reliable model to investigate pathogenetic mechanisms in ALS; indeed, the molecular alterations that we have highlighted in these cells nicely mirror those reported in neuron cells. In this context, our proteomics data from fibroblasts further support the possibility of identifying candidate therapeutic targets among proteins involved in general protein homeostasis.

Dysfunctional protein homeostasis and accumulation of protein inclusions in neuronal cells of ALS patients have been recently demonstrated by several studies; however, it is still unclear whether these mechanisms are causal in the neurodegenerative process or represent the “symptoms”. In this frame, proteomics approaches are central to identifying both primary pathological proteins and those proteins involved in downstream events. Several proteomics platforms have been applied to ALS models to uncover pathological mechanisms and to perform biomarker discovery, as recently summarized by Hedl and colleagues [31]. In particular, Narayan and coworkers applied a SILAC approach to quantitatively analyze the proteome of fibroblasts from ALS patients and matched controls, thus identifying 33 proteins differentially regulated, among which ApoB48, Hsp20 and Fibulin-1 were proposed as novel biomarkers and therapeutic targets [32]. In another recent study, a tandem mass tag (TMT) approach was used to analyze the proteome of fibroblast-derived iPSCs in order to validate genome-wide RNA instability in ALS and FTD patients [33]. In this context, our proteomics analysis of fibroblasts is the first one reporting a comparison between ALS patients carrying the *C9ORF72* repeat expansion and ALS patients who tested negative for it, thus specifically investigating *C9ORF72*-related pathogenetic mechanisms in ALS.

The generation of a PPI network including all differentially expressed proteins allowed us to highlight a significant main component of highly interconnected nodes (129 out of 216 nodes; *p* value < 1 × 10^−4^) to be used as input for a new functional enrichment analysis. This network-based strategy was crucial to unveil additional *C9ORF72*-related pathways, thanks to the exclusion of unconnected proteins. As expected, the ORA of the proteins of the main component further highlighted “proteostasis” as a crucial enriched term (translation initiation and elongation factors, members of the HSP90 family, proteasome 26S subunits, ribosomal proteins, aminoacyl-tRNA synthetases, SRP receptor subunits) but also unveiled nuclear and mitochondrial transports (RAN and RAN-GTPase-activating proteins, SLC25A5) and vesicle trafficking (VPS4B). Moreover, by the fragmentation of the main component of the PPI network into sub-communities, other pathways emerged from the enrichment analysis, such as signal transduction (STAT1), mitochondrial processes including TCA cycle, respiratory electron transport and ATP synthesis (COX2, COX5A, VDAC1) and the ER-phagosome pathway. Strikingly, these pathways (i.e., nuclear and mitochondrial transports, vesicle trafficking, mitochondrial bioenergetics and the ER-phagosome network) are new in the context of *C9ORF72*-mediated pathogenesis of ALS, thus demonstrating that our network-based strategy was a powerful tool to suggest candidate molecular mechanisms to be further investigated in ALS. In keeping with our results, altered bioenergetics has been reported in fibroblasts from ALS patients carrying the *C9ORF72* repeat expansion [34]. Moreover, Mehta and colleagues recently demonstrated that MNs obtained from patient-derived iPSCs (*C9ORF72*-mutated ALS patients) have shorter axons, impaired axonal transport of mitochondrial cargos and altered mitochondrial bioenergetics [35]. RNAseq also revealed reduced gene expression of mitochondrially encoded electron transport chain transcripts and the analysis of autoptic samples confirmed the selective dysregulation of such mitochondrial transcripts in ventral horn spinal MNs [35]. In addition, there is growing evidence from in vitro and in vivo models of *C9ORF72*-related ALS supporting a crucial role in pathogenesis for endoplasmic reticulum stress (which activates the unfolded protein response, UPR) and mitochondrial dysfunction. In particular, the disruption in the signaling between the ER and mitochondria through calcium ions has been proposed as a trigger for mitochondrial dysfunction and apoptosis, constituting a candidate therapeutic target in ALS [36]. Eventually, the functional enrichment analysis of the main component of the PPI network also led us to the identification of a peculiar pathway, which has never been associated with *C9ORF72*-mediated pathogenesis and is still poorly investigated and understood, i.e., the Slit/Robo signaling pathway (see Supplementary Table S6). Slit proteins are secreted glycoproteins which interact with Robo transmembrane receptors, thus regulating several processes in different cell types, such as neuronal axon guidance, cell proliferation, cell migration and angiogenesis [37]. Robo therapy with monoclonal antibodies (mAb) is currently under investigation to treat cancer and vascular diseases. However, Slit/Robo signaling is also crucial for axonal development and radial migration of neurons [38,39,40]; therefore, this pathway is also attracting increasing interest as a possible target in the treatment of neurological disorders.

Lastly in our experimental workflow, we also analyzed the proteome of skin fibroblasts from two *C9ORF72*-mutated FTD patients and compared them with the proteomics profiles of ALS patients. Due to small sample size, we preferred the use of a supervised multivariate approach instead of a differential expression analysis so as to try and identify a combination of features that was able to separate the three groups (FTD_C9, ALS_C9 and ALS_N). This last comparison allowed us to highlight as discriminating features (i.e., separating FTD from ALS patients) some proteins involved in mitochondrial dynamics and vesicle trafficking. This result represents a proof of concept that disease-specific (ALS vs. FTD) and *C9ORF72*-specific (ALS_C9 and FTD_C9 vs. ALS_N) proteomics signatures might be identified through this analysis, by increasing the number of FTD patients involved in the study.

## 4. Materials and Methods

### 4.1. Patients Recruitment, Skin Biopsy and Primary Fibroblast Cell Lines

Three groups of patients were recruited for this study (Table 1): (i) ALS_C9, i.e., ALS patients carrying the solely *C9ORF72* repeat expansion (*n* = 8; five males and three females; mean age 59); (ii) ALS_N, i.e., ALS patients negative for mutations in the main causative ALS genes (*C9ORF72, SOD1, TARDBP* and *FUS*) or carrying mutations other than the *C9ORF72* repeat expansion (*n* = 8; four males and four females; mean age 55); and (iii) FTD_C9, i.e., FTD patients carrying the *C9ORF72* repeat expansion (*n* = 2; two males; mean age 60). Full thickness skin biopsies were obtained from deltoid through the punch technique under local anesthesia after obtaining patient informed consent. This study was approved by the local Ethics Committee (“Comitato Etico interaziendale Novara”, protocol name: PATSLA, code: CE 54/17, approved on 3 April 2017). The investigations were carried out following the rules of the Declaration of Helsinki of 1975 revised in 2013 (https://www.wma.net/policies-post/wma-declaration-of-helsinki-ethical-principles-for-medical-research-involving-human-subjects/ (accessed on 15 July 2018)).

### 4.2. Cell Culture

Primary skin fibroblast cell lines were isolated from skin biopsies as follows. Skin specimens were washed with 70% ethanol and physiological solution and incubated with 2 mg/mL Dispase II (Merck KGaA, Darmstadt, Germany) overnight at 4 °C. Then, the dermis was separated using sterile tweezers, cut into 2–4 mm pieces and plated on a 6-well microplate. A squared sterile glass was put above and 1 mL of high glucose Dulbecco’s modified Eagle’s medium, DMEM (Euroclone, Milan, Italy) supplemented with 20% fetal bovine serum, FBS (Euroclone, Milan, Italy) was added to each well. After 3 weeks, dermis and glasses were removed and fibroblasts were detached with 0.25% Trypsin and 0.02% EDTA (Euroclone, Milan, Italy) and plated in 25 cm^2^ flasks. Cells were then cultured in DMEM supplemented with 15% (*v*/*v*) FBS, 100 U/mL penicillin, 100 mg/mL streptomycin (Euroclone, Milan, Italy) and 2 mM L-glutamine (Euroclone, Milan, Italy) and maintained at 37 °C under humidified conditions and 5% CO_2_. Cells were sub-cultured twice weekly, detached with Accutase (Euroclone, Milan, Italy) and centrifuged at 500× *g* for 10 min at room temperature. Cells were used for proteomics analysis at passage number lower than 10. Cell pellets were collected, washed in PBS, frozen in liquid nitrogen and stored at −80 °C.

### 4.3. Quantitative Proteomics by LC-MS/MS

Whole cell pellets were lysed in RIPA buffer (50 mM Tris-HCl pH 8.0; 5 mM EDTA pH 8.0; 250 mM NaCl; 1% Triton X-100; 0.1% SDS; 0.25% sodium deoxycholate) plus 1% *v*/*v* protease inhibitor cocktail (Merck KGaA, Darmstadt, Germany) and protein content was quantified by Bradford assay (Bio-Rad Laboratories, Hercules, CA, USA). Thirty micrograms of proteins for each sample was transferred to a Microcon-10 centrifugal filter with 10 kDa MWCO (Merck KGaA, Darmstadt, Germany) and processed following a digestion protocol previously described [41]. Briefly, proteins were denatured upon filter-aided buffer exchange to urea buffer (UB: 8 M urea, 100 mM Tris-HCl, pH 8.5) and subsequently reduced in 8 mM DTT in UB (15 min at 56 °C) and alkylated in 0.05 M iodoacetamide in UB (20 min at RT). All the samples underwent proteolytic digestion by trypsin enzyme upon filter exchange to 0.05 M ammonium bicarbonate (AMBIC) solution, using a protease:protein ratio of 1:50 (*w*/*w*), overnight at 37 °C. Digestions were blocked by adding formic acid (FA) to a final concentration of 0.2% (*v*/*v*) and the peptides were recovered from the filter in 0.05 M AMBIC, concentrated in a speedvac and stored at −80 °C until use. Digested peptides were then diluted in a solution of 0.1% FA and 3% acetonitrile (ACN) in order to load 0.25 ng of each sample, spiked with 100 fmol of EColiClpB Hi3 standard (Waters Corporation, Milford, MA, USA) on a symmetry C18 trap column, 100 Å, 5 µm, 180 µm × 20 mm, (Waters Corporation, Milford, MA, USA). Peptides were thus separated by a 125 min reverse phase gradient at 1.2 µL/min (linear gradient, 2–40% ACN over 90 min) using an HSS T3 100 Å 1.8 µm, 150 µm × 100 mm iKey (Waters Corporation, Milford, MA, USA) maintained at 40 °C on an ACQUITY M class UPLC system (Waters Corporation, Milford, MA, USA). Separated peptides were analyzed in a shotgun experiment on a Synapt G2-S*i* mass spectrometer (Waters Corporation, Milford, MA, USA) directly coupled to the chromatographic system. Data were acquired in high definition MS^E^ (HDMS^E^), a data-independent acquisition (DIA) protocol where ion mobility separation (IMS) has been integrated into LC-MS^E^ workflow [42]. Mass spectra were acquired in positive polarity and resolution analyzer mode. TOF MS was operating over 50–2000 *m*/*z* using a scan time of 0.5 s and a continuum data format. Data were post-acquisition lock mass corrected using the doubly charged monoisotopic ion of (Glu1)-Fibrinopeptide B (Waters Corporation, Milford, MA, USA) sampled every 30 s. For IMS, wave height at 40 V, wave velocity of 1000 m/s and transfer wave velocity of 175 m/s were applied. Instrument settings were defined to apply a drift time specific transfer collision energy ramp as previously described [27]. Data from two replicate experiments for each sample were processed for qualitative and quantitative analysis using the Progenesis QI for Proteomics v4.1 software (Waters Corporation, Milford, MA, USA). The qualitative identification of proteins was obtained by searching in human database (Uniprot 2019_11, restricted to *Homo sapiens* taxonomy). Search parameters were set as: 6 ppm for peptide tolerance and 10 ppm for fragment tolerance, minimum one fragment ion matched per peptide, minimum three fragment ions matched per protein, minimum one peptide matched per protein, one missed cleavage accepted, carbamidomethylation of cysteines as fixed modification and oxidation of methionines as variable modification and false discovery rate (FDR) of the identification algorithm at 1%. Quantifications for all identified proteins are reported in Supplementary Table S1. Label-free quantitative analysis was obtained by using the “absolute quantification using HiN” option integrated in the software [43] that averaged the most abundant n peptides (*n* = 3) for each protein to provide a reading for the protein signal. The Hi3 *E. coli* standard (Waters Corporation, Milford, MA, USA) was used as reference. The expression analysis was performed considering the technical replicates for each experimental condition, following the hypothesis that each group is independent. Under this quantification scheme, 1198 quantifiable proteins have been included.

The mass spectrometry proteomics data have been deposited in the ProteomeXchange Consortium via the PRIDE [44] partner repository with the dataset identifier PXD023866 and DOI 10.6019/PXD023866.

Protein quantifications were averaged feature-wise per technical replicate to obtain one column per sample in the raw quantification table. Protein amounts were normalized using the normalize.quantiles function implemented by preprocessCore package [45]. Differential expression was performed by fitting a linear model over the log-transformed normalized level of expression. The coefficients for each feature were extracted for the comparison ALS_C9 vs. ALS_N. *p* values were adjusted for multiple testing using the Benjamini–Hochberg (BH) correction. Thresholds for significance were set to FDR ≤ 0.05 (≥1.3 in −log_10_ scale) and |log_2_(FC)| ≥ 0.3 (equal to 1.23-fold increase/decrease). Volcano plots were generated with ggplot2 [46]. Heatmaps were generated using the R package ComplexHeatmap [47]. The matrix used contained the normalized expression values for the significant features after z-score normalization (feature-wise).

Over-representation analysis was performed using WebGestalt 2019 (http://www.webgestalt.org/ (accessed on 10 June 2021)), where “genome protein-coding” was used as reference set for Fisher’s test and either “Reactome” or “KEGG” was used as the functional database (https://reactome.org/ (accessed on 10 June 2021) and https://www.genome.jp/kegg/ (accessed on 10 June 2021)).

### 4.4. Network Analysis

All significantly altered proteins were considered for network construction and functional enrichment analysis. The protein–protein interaction network was generated and visualized using Cytoscape 3.8.2 [48]. The public database IMEx (https://www.imexconsortium.org/ (accessed on 20 July 2021)) was queried through Cytoscape using the Proteomics Standard Initiative Common QUery InterfaCe (PSICQUIC) standard. The network was filtered for human proteins to remove homology inferences. All self-loops and duplicated edges were removed. The main component of the PPI network thus generated was used to perform an over-representation analysis using WebGestalt 2019 (http://www.webgestalt.org/ (accessed on 20 July 2021)), where “genome protein-coding” was used as reference set for Fisher’s test and “Reactome” was used as the functional database (https://reactome.org/ (accessed on 20 July 2021)). Clusters were built with the GLay community-finding algorithm [49]. The GLay environment provides layout algorithms optimized for large networks and allows the exploration and analysis of highly connected biological networks. Proteins included in the sub-clusters were analyzed by ORA as described above.

Voronoi plot were generated using Proteomaps (https://proteomaps.net/ (accessed on 30 June 2021)) [50]. The input was a table of the significant features. The size of the tile was mapped to the −log_10_(FDR).

### 4.5. Supervised Multivariate Analysis

Multivariate analysis for the classification of the subjects included in the three experimental groups was performed by supervised partial least squares discriminant analysis (sPLS-DA) using the R package mixOmics [51]. The parameter tuning for both the number of components and features was performed using leave-one-out cross-validation method, following the recommended guidelines.

All data analysis was written using the R environment for statistical computing [52].

## 5. Conclusions

Herein, we investigated for the first time the proteome of ALS fibroblast cells in the context of the *C9ORF72* repeat expansion, thus demonstrating that the presence of this genetic variant affects the proteome of these cells. Moreover, we demonstrated that the molecular pathways altered in fibroblasts nicely mirror the main alterations observed in *C9ORF72*-mutated neuron cells, such as dysfunctional protein homeostasis. This evidence supports the use of fibroblasts as an experimental model to study the pathogenesis of ALS. In addition, proteostasis alteration represents a hallmark of neurodegeneration and it is attracting increasing interest as a potential therapeutic target [53]. Eventually, by means of a network-based strategy, we unveiled novel pathways involved in *C9ORF72*-mediated pathogenesis: nuclear and mitochondrial transports, vesicle trafficking, mitochondrial bioenergetics, glucose metabolism, ER-phagosome crosstalk and Slit/Robo signaling pathway. These processes might be further explored to deepen the knowledge about the role of the *C9ORF72* repeat expansion in neurodegeneration and to find candidate disease biomarkers and therapeutic targets.

## Figures and Tables

**Table 1:** Outline of primary skin fibroblast samples from ALS and FTD patients.

Subject	Group	Age at Biopsy	Sex	Cell Passage	Mutation	Note
A610	ALS_C9	69	M	6	*C9ORF72* expansion	/
A670	ALS_C9	64	M	4	*C9ORF72* expansion	/
A863	ALS_C9	45	M	4	*C9ORF72* expansion	/
A899	ALS_C9	52	F	5	*C9ORF72* expansion	/
9848	ALS_C9	57	F	3	*C9ORF72* expansion	Dementia
10008	ALS_C9	59	M	3	*C9ORF72* expansion	/
10879	ALS_C9	80	M	4	*C9ORF72* expansion	Familial history of FTD
10881	ALS_C9	52	F	4	*C9ORF72* expansion	Familial history of FTD
2284	ALS_N	42	F	3	Neg. four ALS genes	/
2293	ALS_N	50	F	3	Neg. four ALS genes	/
5786	ALS_N	53	M	3	Neg. four ALS genes	/
8729	ALS_N	65	M	2	*Fus* 3′-UTR (c.87 C > G)	/
8873	ALS_N	53	M	3	*SOD1* ex 5 p.I149T	/
9718	ALS_N	49	F	3	*SOD1* ex 5 p.L145F	/
9799	ALS_N	70	M	4	*SOD1*, intron 1	Schizophrenia
9847	ALS_N	57	F	3	Neg. four ALS genes	/
10234	FTD_C9	57	M	3	*C9ORF72* expansion	Dementia
10946	FTD_C9	64	M	8	*C9ORF72* expansion	Dementia

## Data Availability

The mass spectrometry proteomics data have been deposited in the ProteomeXchange Consortium via the PRIDE partner repository with the dataset identifier PXD023866 and 10.6019/PXD023866. Project Name: *C9ORF72* repeat expansion affects the proteome of primary skin fibroblasts in ALS and FTD. Project accession: PXD023866. Project doi:10.6019/PXD023866. Reviewer account details. Username: reviewer_pxd023866@ebi.ac.uk. Password: bKSe4Y3G.

## Supplementary Materials

Supplementary materials can be found at https://www.mdpi.com/article/10.3390/ijms221910385/s1.

## Abbreviations

ALS	Amyotrophic Lateral Sclerosis
FTD	Frontotemporal Dementia
*C9ORF72*	Chromosome 9 Open Reading Frame 72
PPI	Protein-Protein Interaction
LC-MS/MS	Liquid Chromatography Tandem Mass Spectrometry
sPLS-DA	Sparse Partial Least Squares Discriminant Analysis
ORA	Over-Representation Analysis
BH	Benjamini–Hochberg
